# A Retrospective Cohort Study of Young Women Spontaneously Choosing to Be Vaccinated against HPV: Outcomes from Their First Cervical Cancer Screening Test

**DOI:** 10.3390/v13030486

**Published:** 2021-03-16

**Authors:** Annarosa Del Mistro, Jessica Battagello, Luca Weis, Vittoria Bressan, Vittorio Selle, Mauro Ramigni, Alessandra Dal Zotto, Antonio Maggiolo, Silvia Gori, Helena Frayle, Marco Zappa, Manuel Zorzi, the Consensus Study Veneto Working Group

**Affiliations:** 1 Immunologia e Diagnostica Molecolare Oncologica, Veneto Institute of Oncology IOV-IRCCS, Via Gattamelata, 64, 35128 Padua, Italy; luca.weis.lw@gmail.com (L.W.); silvia.gori@iov.veneto.it (S.G.); helena.frayle@iov.veneto.it (H.F.); 2 Veneto Tumour Registry, Azienda Zero, 35131 Padua, Italy; jessica.battagello@azero.veneto.it (J.B.); manuel.zorzi@azero.veneto.it (M.Z.); 3 Department of Neuroscience (DNS), University of Padua, 35131 Padua, Italy; 4 Local Health Unit Euganea, 35131 Padua, Italy; vittoria.bressan@aulss6.veneto.it; 5 Local Health Unit Serenissima, 30174 Venezia, Italy; vittorio.selle@aulss3.veneto.it; 6 Local Health Unit Marca Trevigiana, 31100 Treviso, Italy; mauro.ramigni@aulss2.veneto.it; 7 Local Health Unit Pedemontana, 36061 Bassano del Grappa, Italy; alessandra.dalzotto@aulss7.veneto.it; 8 Local Health Unit Scaligera, 37121 Verona, Italy; antonio.maggiolo56@gmail.com; 9 Institute for Cancer Research, Prevention and Clinical Network (ISPRO), 50139 Florence, Italy; m.zappa@ispro.toscana.it

**Keywords:** HPV, prophylactic vaccination, cervical cancer screening, CIN2, CIN3, cancer, prevention, age at vaccination

## Abstract

Background: Efficacy for cervical cancer prevention of opportunistic HPV vaccination in post-pubertal girls is lower than in 11-year-olds. Methods: Women born between 1986 and 1992 vaccinated at 15–25 years of age (at least one dose of 4-valent HPV vaccine) and screened at 24–27 years of age were included. Frequency of opportunistic vaccination, overall and by birth cohort, was calculated; screening outcomes were compared between vaccinated and unvaccinated women. Results: Overall, 4718 (4.9%) HPV-vaccinated, and 91,512 unvaccinated, women were studied. The frequency of vaccination increased by birth cohort, ranging between 1.8% and 9.8%; age at vaccination decreased progressively by birth cohort (*p* < 0.0001). Participation in screening was 60.8% among vaccinated, and 56.6% among unvaccinated, women (*p* < 0.0001). Detection rates (DR) for high-grade lesions were lower in vaccinated women (2.11‰ vs. 3.85‰ in unvaccinated, for CIN3+, *p* = 0.24; 0.0‰ vs. 0.22‰ for cancer). The DR of CIN3+ increased with age at vaccination, scoring respectively 0.0‰, 0.83‰, and 4.68‰ for women vaccinated when they were 15–16, 17–20, and 21–25 years old (*p* = 0.17). Conclusions: In comparison to unvaccinated women, higher compliance with cervical cancer screening invitation and lower CIN3+ DR among vaccinated women was observed. Age at vaccination was inversely correlated to vaccination efficacy.

## 1. Introduction

Human papillomaviruses (HPVs) are responsible of epithelial infections that can either regress spontaneously or, less frequently, cause carcinomas and preneoplastic lesions. Persistent infection of the cervix by high-risk types is the cause of invasive cervical carcinoma [1].

The natural history of cervical HPV infection and the long time between initial infection and lesion progression have led to the development of protocols for cervical cancer screening programs. Secondary prevention practice through population-based organized screening started in Italy in the late 1990s, and important decreases in both incidence and mortality have been obtained [2]. The recent introduction of HPV testing for the screening of women older than 30 years has further enhanced the efficacy of screening [3].

Since 2007, prophylactic vaccines for the prevention of HPV infection by specific types have also been used. Three different formulations, targeting high-risk types only (i.e., bivalent (2v) for HPV16/18), or high- and low-risk types (i.e., quadrivalent (4v), for HPV16/18 and HPV6/11; nonavalent (9v) for HPV16/18/31/33/45/52/58 and HPV6/11), have been in place [4]. The highest efficacy is obtained by vaccination before virus exposure [5]. Free-of-charge active campaigns are in place in many industrialized countries; initially only girls aged 11–12 years were targeted, but subsequently boys of the same age were also included [6]. In Italy, the active campaign started in 2007, involving girls born in 1996. Additional campaigns for older girls have been offered in a few regions (Veneto was not among those), while opportunistic vaccination has been available at full or reduced cost.

An efficacy of 95–100% in preventing persistent infections and lesions related to vaccine types has been demonstrated by randomized clinical trials conducted for vaccine authorization [7]. Subsequently, updated analyses of the trials [8], as well as many post-marketing studies have confirmed this high efficacy, and strengthened the influence of age at vaccination [9].

Since vaccination efficacy is type-dependent, vaccinated women are still in need of secondary prevention by cervical screening, but, owing to their reduced risk, less intensive protocols can be applied. In Italy, a national consensus conference defined tailored screening protocols for women vaccinated at 11–12 years (i.e., screening to start at 30 years of age, use of HPV testing) and highlighted the need of linkage between vaccination and screening databases, and of additional studies to optimize the screening interval [10].

Here we present the results of a survey among young women of birth cohorts not included in active vaccination campaigns, living in the Veneto region (north-east of Italy). We investigated the frequency of opportunistic HPV vaccination and the outcomes of the first cervical cancer screening round in vaccinated girls, in comparison to non-vaccinated peers.

## 2. Methods

### 2.1. Setting

The Veneto region (4.9 million inhabitants) is divided into nine local health units (LHU) that operate all prevention activities by following national recommendations and prevention plans. Procedures for periodic monitoring are in place for both primary and secondary prevention.

Population-based cervical cancer screening (with call-recall invitations) for women in the 25–64 age group has been active since 1997, the roll out phase being completed by 2002. From 1997 to the first months of 2015, all women were invited to undergo a pap smear every 3 years; thereafter, HPV-DNA testing every 5 years was implemented for 30–64-year-old women, while a pap smear every 3 years was maintained for women aged 25–29 years. The HPV screening protocol included HPV testing with cytology triage for HPV positives (HPV+) [11]; HPV+ women with positive cytology (diagnosis of atypical squamous cells of undetermined significance (ASC-US) or worse, cyto+) underwent immediate colposcopy; women with HPV+ and negative cytology (cyto-) were referred to HPV re-testing at 1 year. Between 2009 and 2015, pilot projects for the applicability of HPV screening were carried out by three LHUs (Euganea, Polesine, and Serenissima) involving 25–64-year-old women [12,13]. Therefore, during the study period both cytology-based and HPV-based screening programs were ongoing in different LHUs.

An active campaign of HPV vaccination for girls born in 1996 was introduced in Veneto in 2008. Vaccination was free-of-charge up to the 18th (and later 25th) birthday for girls born from 1996 onward. 4v and 9v vaccines were used in 2008–mid2017, and from mid2017 onward, respectively. Vaccination coverage of girls ranged between 75 and 82%, with a recent decrease for the last birth cohorts [14]. Women not included in the active-vaccination birth cohorts could choose to get the vaccination for a fee, mostly at a reduced cost that ranged between 104.5 € to 79 € for each dose, in 2009 and 2019, respectively.

The study was performed in the following LHUs: Marca Trevigiana, Serenissima, Euganea, Pedemontana, and Scaligera. Although the COVID-19 pandemic precluded the inclusion of all the LHUs of the region, the study covered about 77% of the population living in Veneto.

### 2.2. Study Design

In this large cohort study, women resident in the study areas who were born between 1986 and 1992, and who had been invited for the first time by the screening program between 2011 and 2017, were identified. Through record linkage with the LHUs’ vaccination databases, we identified the women who underwent opportunistic HPV vaccination, and built two study cohorts: vaccinated and non-vaccinated. We included in the vaccinated cohort all the women: (i) who received at least one dose of vaccine, (ii) who were vaccinated (first dose) before the 26th birthday, (iii) whose vaccination date (first dose) was between January 2008 and December 2017, and (iv) who were vaccinated (first dose) before the date of the first screening invitation. The non-vaccinated cohort included non-vaccinated women and women who were vaccinated after the date of the first screening invitation; the number of the latter group was small (*n* = 491), and accounted for 0.5% (491/91,512) of the entire cohort.

Data about invitation to screening, participation, and screening results were obtained from cervical cancer screening databases. Information on screened women was collected, including a 2-year follow up after the screening test.

### 2.3. Statistical Analysis

The primary outcome of the study was the detection of cervical intraepithelial neoplasia grade three or cervical cancer (CIN3+) among vaccinated and non-vaccinated women, computed as the proportion of women with a CIN3+ lesion detected by the end of the follow up among all women who attended screening. The results of the two cohorts were also compared by calculating the relative risk (RR) along with 95% confidence intervals (CI). Results were reported overall and separately for women invited to cytology or to HPV testing, according to the LHU organization. Vaccinated women were analyzed overall, and stratified according to age at vaccination (<17, 18–20, >20 years).

For women invited to cytology, the following additional indicators were calculated: compliance with invitation; proportion of positive cytology (cyto+) among screened women, overall and by cytology grade (low grade (LG), including atypical squamous cells of undetermined significance (ASC-US) and low-grade squamous intraepithelial lesion (LSIL); or high grade (HG+), including atypical squamous cells-cannot exclude high-grade SIL (ASC-H), high-grade squamous intraepithelial lesion (H-SIL), atypical glandular cells favor neoplastic (AGC), adenocarcinoma in situ (AIS), squamous cell carcinoma, adenocarcinoma); detection of CIN3+ and CIN2+ among screened women; and positive predictive value (PPV) for CIN3+ of cyto+ at colposcopy.

For women invited to HPV testing, the following indicators were reported: compliance with invitation; proportion of positive HPV (HPV+) among screened women; positive triage cytology among HPV+; detection of CIN3+ and CIN2+; and PPV for CIN3+ of HPV+cyto+ at colposcopy.

Differences in outcome distributions among cohorts were tested using the chi-square test (χ^2^), and Fisher’s exact test in case of small sample size.

Statistical significance was set at 0.05 and analyses were computed using SAS, version 9.4 statistical package (SAS Institute, Cary, NC, USA) and R-software environment, version 4.0.2.

### 2.4. Ethics

The Italian legislation identifies cancer registries as collectors of personal data for surveillance purposes without explicit individual consent. The approval of a research ethics committee was not required, since this study is a descriptive analysis of individual data without any direct or indirect intervention on patients.

## 3. Results

### 3.1. Study Cohorts

**Figure 1:**
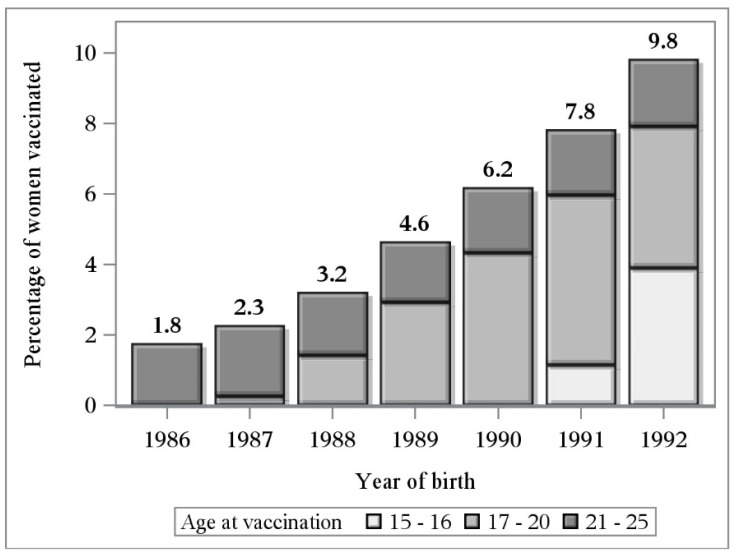
Percentage of vaccinated women according to year of birth, stratified by age at vaccination.

Overall, 96,230 women were included in this study: 4718 HPV-vaccinated and 91,512 unvaccinated (Table 1). Less than five percent of women (4.9%) were HPV-vaccinated (>95% with the 4v vaccine), but the temporal trend of opportunistic vaccination was increasing, and ranged between 1.8% and 9.8% for girls born in 1986 and 1992, respectively (Figure 1). Most women were vaccinated after 17 years of age (88.1%), but age at vaccination decreased progressively by birth cohort (*p* < 0.0001). No vaccination before 17 years of age was observed in women born before 1991, compared respectively with 14.7% and 36.7% of vaccinated women born in 1991 and 1992 (Figure 1).

### 3.2. Compliance with Invitation

Most women were invited to cytology-based screening (84.2%), and only 15.8% to perform an HPV test (Table 1). Compliance was 56.8% overall, 60.8% among vaccinated and 56.6% among non-vaccinated women (*p* < 0.0001), with statistically significant differences both for women invited to perform cytology (62.3% vs. 58.2%; *p* < 0.0001) and HPV testing (53.4% vs. 48.0%; *p* = 0.01). Compliance with first screening invitation was 20.7% higher for cytology protocol than HPV-protocol (*p* < 0.0001).

### 3.3. Overall Detection of CIN3+, CIN2+, and Cancer

Considering all the lesions detected in women screened both with cytology and with HPV test (including follow up), five CIN3+ were diagnosed in the vaccinated cohort and 174 in the non-vaccinated cohort. The detection of CIN3+ was halved in those HPV-vaccinated as compared with non-vaccinated (2.11‰ vs. 3.85‰, RR 0.5; 95%CI 0.2–1.3). However, this difference was not statistically significant (*p* = 0.24). No cancer was found among HPV-vaccinated, while 10 cervical cancers were detected in the non-vaccinated cohort (five adenocarcinoma in situ, four invasive squamous cell carcinomas, and one invasive adenocarcinoma), leading to a detection of 0.22 per 1000 screened women (10/45,190).

Overall, 18 CIN2+ were diagnosed in the HPV-vaccinated cohort (detection equal to 7.6 per 1000 screened) and 529 in the non-vaccinated cohort (11.71 × 1000), leading to a RR of 0.6 (95%CI 0.4–1.0) with borderline statistical significance (*p* = 0.08). 

**Figure 2:**
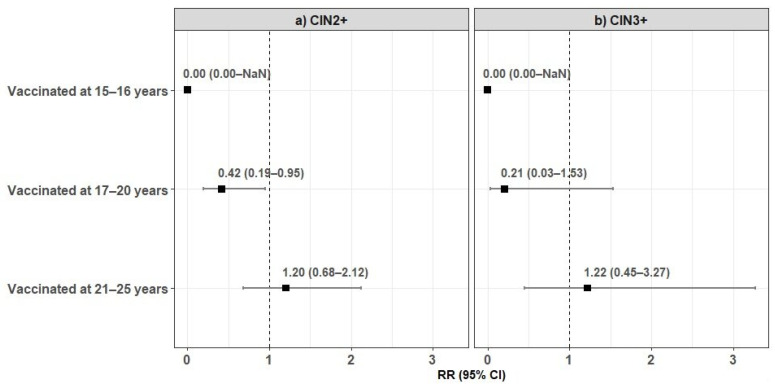
Forest plot of the relative risks of CIN2+ (**a**) and CIN3+ (**b**) in vaccinated vs. unvaccinated women, according to age at vaccination, with 95% confidence intervals. Note: RR: Relative Risk; 95% CI: 95% Confidence Interval; NaN: Not a number.

Age at vaccination affected the number of CIN2+ lesions detected (Table 2). The detection rate increased with age at vaccination, scoring 0, 4.96, and 14.04 per 1000 women vaccinated (*p* = 0.02) when they were 15–16, 17–20, and 21–25 years old, respectively. The five CIN3 cases found among vaccinated women included one woman vaccinated at 17–20 years and four at 21–25 years. Figure 2 shows the relative risks (RR) of CIN2+ and CIN3+ in vaccinated vs. unvaccinated women, in relation to age at vaccination.

### 3.4. Results of Cytology-Based Screening

**Figure 3:**
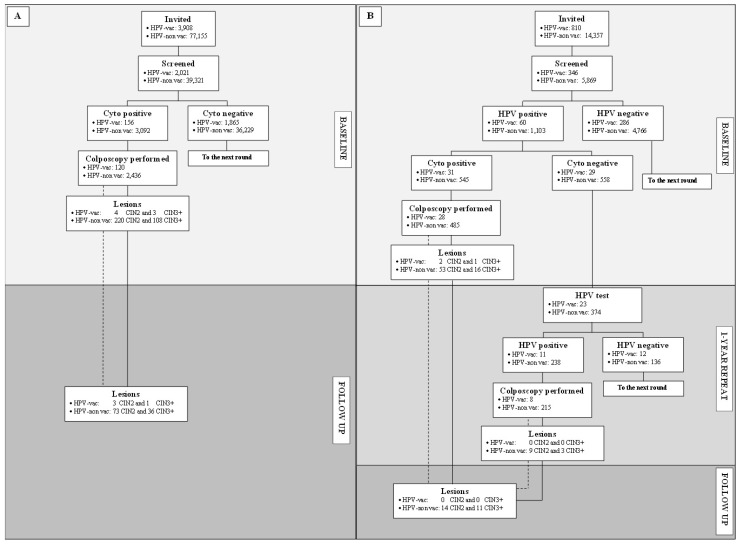
Flowchart of the study for women invited to cytology-based screening (**A**) or to HPV-based screening (**B**).

Overall, 81,063 women were invited to screening with cytology. Among the 41,342 compliers, 2021 were HPV-vaccinated and 39,321 non-vaccinated (Table 3 and Figure 3). A positive cytology was recorded in 7.9% women: 7.7% of HPV-vaccinated and 7.9% of non-vaccinated (*p* = 0.86). A HG+ cytology was diagnosed in 0.6% (13/2,021) of HPV-vaccinated vs. 0.7% (273/39,321) of non-vaccinated women (*p* = 0.89). Among the 2715 women who were referred to colposcopy, compliance was 90.9% and 94.3% for HPV-vaccinated and non-vaccinated, respectively. The percentage of women who did not perform colposcopy because they reported that they had undergone a non-programmatic examination outside the screening centers was higher for HPV-vaccinated compared to non-vaccinated women (6.8% vs. 4%; *p* = 0.11). The detection rate of CIN3+ among cyto+ women was 2.68‰ (111/41,342) overall; 1.48‰ among HPV-vaccinated (3/2021) and 2.75‰ for non-vaccinated women (108/39321; *p* = 0.4) (Table 3). The PPV for CIN3+ at colposcopy was 4.3% (111/2556) overall; 2.5% (3/120) in HPV-vaccinated and 4.4% (108/2436) in non-vaccinated women (*p* = 0.49).

During follow-up, one CIN3+ was diagnosed in HPV-vaccinated and 36 in non-vaccinated women. Considering all the lesions detected by the end of the follow up, the relative risk of CIN3+ for HPV-vaccinated was lower, although statistically non-significant (RR 0.5; 95%CI 0.2–1.5; *p* = 0.33).

### 3.5. Results of HPV-Based Screening

Overall, 15,167 women were invited to screening with HPV test. Among the 6215 compliers, 346 were HPV-vaccinated and 5869 non-vaccinated (Table 3 and Figure 3). Overall HPV positivity was 18.7%: 17.3% for HPV-vaccinated and 18.8% for non-vaccinated (*p* = 0.54). Among HPV positives, triage cytology was positive in 51.7% (31/60) in the HPV-vaccinated and 49.4% (545/1103) in the non-vaccinated. Overall, 576 women were referred to colposcopy with a compliance of 90.3% and 90.0% for HPV-vaccinated and non-vaccinated, respectively. The detection rate of CIN3+ was 2.74‰ (17/6216) overall, and similar for HPV-vaccinated (2.89‰) and non-vaccinated (2.73‰; *p* = 1). The PPV for CIN3+ at colposcopy was 3.3% overall, 3.6% in the HPV-vaccinated and 3.3% in the non-vaccinated (*p* = 1).

Among HPV+cyto- women who repeated the HPV test after one year, HPV positivity was non-significantly lower for HPV vaccinated than for non-vaccinated (47.8% vs. 63.6%; *p* = 0.51) (Figure 3). At one-year repeat, three CIN3+ lesions were found, all in the non-vaccinated group, and at follow-up three further CIN3+ were detected, again only in non-vaccinated. Considering the cumulative lesions detected at the baseline, at 1-year repeat and at follow-up, the relative risk of CIN3+ for HPV-vaccinated was lower, although the result was not statistically significant (RR 0.6; 95% CI 0.1–4.1; *p* = 1).

## 4. Discussion

Cervical cancer prevention relies on the integrated use of HPV vaccination and screening. Vaccinated women will need to continue screening but, owing to the lower cancer risk conferred by vaccination, their screening protocols need to be modified. Our study was nested within a multicenter project aimed at evaluating the screening outcomes in women vaccinated within organized campaigns or as the result of a spontaneous decision, in order to gain data useful to define how to modify the screening protocols (age at first screening, primary screening test, screening intervals) for vaccinated women. We performed a survey on young women who spontaneously chose to be vaccinated at 15–25 years of age, and found that the frequency of vaccination increased over time (from 2% in the 1986 cohort, up to 10% in the 1992 cohort) and that vaccinated women had a lower detection of high-grade lesions than their unvaccinated peers (CIN3+: 2.11‰ vs. 3.85‰, respectively, with 0 vs. 10 cases of carcinoma; CIN2+: 7.6‰ vs. 11.71‰, respectively). However, the efficacy was inversely associated with age at vaccination. Our data are in line with those of other studies [5,9,15], notwithstanding that vaccination was opportunistic and for a fee in our study, while it was free-of-charge and opportunistic [5], or free-of-charge and organized as a catch-up cohort [15] in the other areas. The different vaccination accessibility influenced both the number and the socio-demographic characteristics of the vaccinated subjects included in the studies, but vaccination before age 17 demonstrated a significantly higher protection than vaccination at older ages in all studies. Efficacy was lower than that observed for women vaccinated at 11 years, thus reinforcing the importance of promoting HPV vaccination in young girls; interestingly, in our study population, age at opportunistic vaccination decreased over time by birth cohort.

Compliance with screening invitation was about 4% higher among vaccinated than unvaccinated women; higher uptake among vaccinated women was also reported in other studies [16,17]. Since screening must continue in vaccinated women to ensure individual protection, this is an important finding. Nonetheless, we cannot exclude that these women might constitute a cohort attentive to health and prevention practices and with good access to health services, as already suggested [16,17]. The design of the study precluded the collection of sociodemographic data, but the registration of a higher percentage of recent (non-programmatic) pap smears and colposcopies among vaccinated than unvaccinated women seems to support this hypothesis. Moreover, these women may also have a different risk of HPV exposure, but no data are available for our study cohorts.

In comparison to unvaccinated women, the detection of CIN3+ and of CIN2+ decreased by 50% and 60%, respectively, in vaccinated women. Such differences did not reach statistical significance (*p* = 0.24 and *p* = 0.08, respectively), most probably due to the low number of events (especially of CIN3 lesions) in the vaccinated group. A similar result was observed for the frequency of abnormal cytology in another Italian study, where the number of women in the two study cohorts was similar [15]. Efficacy was associated with age at vaccination, with a statistically significant difference in CIN2+ detection between women vaccinated before 21 years and those vaccinated later or non-vaccinated. In particular, no lesions were found among women vaccinated when they were 15–16. Indeed, the age 17 has been repeatedly reported as a significant cutoff, probably in relationship with sexual debut [5,9,18]. These observations also indicate that HPV vaccination holds a role after age 12, but highlights the importance of promoting it before age 17. In this regard, school involvement can play a very important role, by increasing knowledge, attitudes, and awareness, possibly integrated with other strategies [19].

The great majority of the women included in our study were vaccinated with the 4vHPV vaccine; since the 9vHPV vaccine potentially further expands, by 75% or more, the protective efficacy for high-grade preneoplastic cervical lesions [20], it might be speculated that its use will increase the cost:benefits of cervical screening in vaccinated women.

While women vaccinated in their 12th year may start screening when they are 30 years old [10], our study did not produce convincing evidence that women vaccinated at 15–25 years of age may postpone their screening start as well. The subgroup of women vaccinated before the age of 17 could represent an exception, which needs to be confirmed by larger studies.

Differently from other studies [21], the positive predictive values at colposcopy were similar among vaccinated women who were screened with cytology or with HPV test. Therefore, notwithstanding that the detection of CIN3+ was higher among women screened with HPV test, our results do not confirm the indication of the Italian Consensus Conference to use HPV testing for screening women vaccinated in their 12th year [10]. However, the number of vaccinated women screened by HPV testing in our study was very small. An economic assessment of the use of HPV vs. cytology testing predicted that screening effectiveness was 5% higher by the former strategy in both unvaccinated and vaccinated women, and that costs would be 26% and 19% lower for screening cohorts of women who had been offered or not offered vaccination [22]. Interestingly, a lower PPV of cytology for CIN2+ was found among vaccinated women in Sweden, with a stronger decrease for those vaccinated at younger ages [23].

The strengths of our study are the high coverage (77%) of the population living in the Veneto region, and the use of both screening protocols (i.e., cytology and HPV testing). The results add new information on the preventive attitudes of girls and women, and on the efficacy of opportunistic vaccination. On the other hand, the relatively low representation of vaccinated women (about 5%) in the study population and the absence of data on sociodemographic characteristics limit the generalizability of our findings.

In conclusion, our survey indicates that implementation of HPV vaccination among adolescents, particularly before age 17, plays an important role in the prevention of cervical cancer (and possibly of other HPV-driven cancers).

## Figures and Tables

**Table 1:** Distribution of women among study cohorts (HPV-vaccinated vs. HPV-non-vaccinated) and compliance with screening invitation.

	HPV Vaccinated	HPV Non-Vaccinated	All	*p*-Value ^1^
Women invited	4718	91,512	96,230	
Screening test proposed, *n* (%) Cytology HPV	3908 (82.8) 810 (17.2)	77,155 (84.3) 14,357 (15.7)	81,063 (84.2) 15,167 (15.8)	
Screened women, *n* (%)	2367 (50.2)	45,190 (49.4)	47,557 (49.4)	0.3
Undelivered invitations, *n* (%)	31 (0.7)	1248 (1.4)	1279 (1.3)	<0.0001
Not screened: referred a recent cytology, *n* (%)	661 (14)	8092 (8.8)	8753 (9.1)	<0.0001
Crude ^2^ compliance, %	50.5	50.1	50.1	0.57
Adjusted ^3^ compliance, % Cytology HPV	60.8 62.3 53.4	56.6 58.2 48	56.8 58.4 48.3	<0.0001 <0.0001 0.01

^1^ Chi-squared test. ^2^ Screened women/(invited women—undelivered invitations). ^3^ Screened women/(invited women—undelivered invitations—women excluded after invitation).

**Table 2:** HPV-vaccinated women: comparison of the main results according to age at vaccination.

Age at Vaccination (Years)	15–16	17–20	21–25	*p*-Value ^1^
Number of screened women	302	1210	855	
CIN2 × 1000 screened women (*n*)	0 (0)	4.13 (5)	9.36 (8)	0.12
*p*-value ^2^	0.18	0.18	0.56	
CIN3+ × 1000 screened women (*n*)	0 (0)	0.83 (1)	4.68 (4)	0.17
*p*-value ^2^	0.63	0.1	0.58	
CIN2+ × 1000 screened women (*n*)	0 (0)	4.96 (6)	14.04 (12)	0.02
*p*-value ^2^	0.05	0.03	0.52	

^1^ Fisher exact test. Comparison within the vaccinated group. ^2^ Fisher exact test. Comparison with non-vaccinated women.

**Table 3:** Women screened by cytology or HPV protocol: comparison of the main results, according to vaccination status.

	HPV Vaccinated	HPV Non-Vaccinated	All	Relative Risk (95% CI) ^1^	*p*-Value ^2^
***Cytology-Based Screening***					
Women screened	2021	39,321	41,342		
Positive cytology % (*n*)	7.7 (156)	7.9 (3092)	7.9 (3248)	1.0 (0.8–1.1)	0.86
Low grade cytology % (*n*)	7.1 (143)	7.2 (2819)	7.2 (2962)	1 (0.8–1.2)	0.91
High grade cytology % (*n*)	0.6 (13)	0.7 (273)	0.7 (286)	0.9 (0.5–1.6)	0.89
CIN3+ detection ‰ at baseline * (*n*)	1.48 (3)	2.75 (108)	2.68 (111)	0.5 (0.2–1.7)	0.4
CIN2+ detection ‰ at baseline * (*n*)	5.44 (11)	8.34 (328)	8.2 (339)	0.7 (0.4–1.2)	0.2
PPV for CIN3+ % (CIN3+ / colposcopies)	2.5 (3/120)	4.4 (108/2436)	4.3 (111/2556)	0.6 (0.2–1.7)	0.49
CIN3+ detection ‰ overall ° (*n*)	1.98 (4)	3.66 (144)	3.58 (148)	0.5 (0.2–1.5)	0.33
***HPV Test-Based Screening***					
Women screened	346	5869	6215		
Positive HPV % (*n*)	17.3 (60)	18.8 (1103)	18.7 (1163)	0.9 (0.7–1.2)	0.54
Positive triage cytology among HPV+ % (*n*)	9 (31)	9.3 (545)	9.3 (576)	1 (0.7–1.4)	0.91
CIN3+ detection ‰ at baseline * (*n*)	2.89 (1)	2.73 (16)	2.74 (17)	1.1 (0.1–8.0)	1
CIN2+ detection ‰ at baseline * (*n*)	8.67 (3)	11.76 (69)	11.58 (72)	0.7 (0.2–2.3)	0.8
PPV for CIN3+ % (CIN3+ / colposcopies)	3.6 (1/28)	3.3 (16/485)	3.3 (17/513)	1.1 (0.1–7.9)	1
CIN3+ detection ‰ overall ° (*n*)	2.89 (1)	5.11 (30)	4.98 (31)	0.6 (0.1–4.1)	1

* baseline: lesions detected after cyto+ (cytology-based screening) and after HPV+cyto+ (HPV-based screening). ° overall: including lesions detected during follow up (and, in the HPV-based screening, at 1-year repeat of HPV+cyto- women). Abbreviations: CIN2, CIN3+, CIN2+ = cervical intraepithelial neoplasia grade 2, 3+, 2+; HPV = human papillomavirus; ASC-US = atypical squamous cells of undetermined significance; PPV = positive predictive value. ^1^ The reference is: HPV non-vaccinated cohort. ^2^ Chi-squared test or Fisher exact test.

## Data Availability

The data presented in this study are available on request from the corresponding author. The data are not publicly available due to privacy policies.
